# Patients’ Perspectives on the Implementation of AI in Radiological Diagnostics: Focus Group Study

**DOI:** 10.2196/89178

**Published:** 2026-05-25

**Authors:** Cornelia R Karger

**Affiliations:** 1 Brain and Behaviour/ Research Group: Neuroethics and Ethics in AI, Institute of Neurosciences and Medicine, Forschungszentrum Jülich GmbH, Wilhelm-Johnen-Strasse, Jülich, 52425, Germany, 49 2461612794

**Keywords:** artificial intelligence, AI, radiology, diagnostics, trust, acceptance, focus groups, qualitative study

## Abstract

**Background:**

Rapid developments in artificial intelligence (AI) will enable its widespread use in radiological diagnostics in the near future. Patients will then be confronted with findings generated with the help of AI. Understanding patients’ perspectives on the use of this technology is one of the key factors for its successful implementation.

**Objective:**

This qualitative study aimed to gain insight into patients’ reasoning about the opportunities and risks of using AI in radiological diagnosis, to identify prerequisites for its acceptance, and to identify aspects that can promote trust in AI diagnoses, especially in scenarios of high personal concern.

**Methods:**

A total of 7 focus groups were conducted with 34 patients (n=15, 44% female participants) aged between 23 and 85 years (mean 49.06, SD 17.08 y), recruited using purposive sampling strategies. Each focus group was audiotaped, transcribed, and analyzed using the method of structured qualitative content analysis.

**Results:**

Study findings show that patients are open to the use of AI in radiological diagnostics. The basic prerequisites for this are (1) scientific evidence of safe outcomes that are more accurate and faster than those without AI; (2) recognizable added value in patient care; (3) transparency in the use of AI and disclosure to the patient; (4) comprehensive, binding measures for quality assurance; and (5) the use of AI solely to support the physician. However, the results indicate that further criteria are important for patients to be willing to choose a radiologist who uses AI and to trust AI diagnoses. In situations where they are personally affected, patients fear that physicians will place too much trust in the AI result and that the physician-patient relationship will become dehumanized. Therefore, the physicians’ abilities and functions that inspire trust from the patients’ perspective must come into play. These include (1) an independent diagnosis by the physician that takes into account not only the clinical context but also the individuality of the patient, (2) a comprehensible explanation of the pros and cons of using AI for patients and clear communication of AI output, and (3) a humane and empathetic physician-patient relationship, which shows that the physician continues to feel responsible for the patient.

**Conclusions:**

The results of the study underscore that a high quality of the entire “sociotechnical” system is an essential prerequisite for patient acceptance of the use of AI in radiological diagnostics and for trust in AI diagnoses. The further development of AI performance must go hand in hand with the creation of framework conditions for its use that meet patients’ expectations of the role of the physician and ensure a trust-building physician-patient relationship. The study provides valuable insights into how such integration of AI into radiological practice can be achieved.

## Introduction

The rapid progress of AI technology has revolutionized health care in various fields [1]. Radiology is among the pioneers of this development. X-ray images, as well as magnetic resonance imaging (MRI) and computed tomography scans, have long been digitized, thus providing a solid basis for integrating AI in the diagnosis, prediction, or classification of diseases [2]. One of the biggest advantages is enhancing the efficiency of interpreting medical images and increasing diagnostic accuracy [3,4].

Studies revealed that AI models can classify lung nodules on computed tomography scans with high accuracy [5,6]. A review of 38 studies, including 2924 cases of multiple sclerosis and 2509 healthy controls reported in 29 studies, showed a sensitivity ranging from 77% to 100% and a specificity of 74% to 100%. Furthermore, 34 studies reported accuracy ranging from 81% to 100% [7]. Comparisons show that the accuracy of AI often matches or even exceeds the performance of radiologists [8,9]. The detection of COVID-19 pneumonia in chest radiography using deep learning algorithms, for example, was achieved with a sensitivity and specificity of 96% and 64%, respectively, compared to radiologists’ sensitivity and specificity, which ranged between 50% and 73% [10]. In patients with amyloid–related imaging abnormalities, radiologists had significantly better detection performance in the assisted setting with a deep learning method (87%) compared to the unassisted setting (71%) [11].

However, there are challenges that must be taken into account when introducing the technology into regular clinical practice.

One key challenge concerns the data used to train AI. The problem is not only the quantity and quality of the training data but, above all, the appropriate validation of the AI model. Critical issues include, for example, intentional or unintentional distortions due to a lack of representativeness or biases in data labels and misclassifications. Most AI applications are developed and trained using datasets from high-income countries. Minority groups and non-Western populations are, therefore, often underrepresented. Such distortions can result in suboptimal performance for certain patient groups and incorrect diagnoses [12-14]. Moreover, the problem of the “black box” nature of AI models makes it difficult to detect and clarify errors. In practice, however, it has so far been the responsibility of radiologists to monitor the output. Therefore, efforts are being made to make deep learning models explainable so that radiologists can fulfill their responsibility [15,16]. At the same time, studies on automation bias show that there is a risk that radiologists may place too much trust in AI results [17].

Beyond the performance of AI, successful implementation depends on the acceptance and trust of those affected, particularly patients [18,19]. Trust is crucial in almost any type of situation where uncertainty exists or undesirable outcomes are possible [20]. In the sensitive field of radiological diagnostics, which carries potentially life-threatening consequences, trust is especially challenged. Acceptance and trust are shaped not only by individual factors, but also by the national context [21]. Unlike people in emerging economies and non-Western countries, individuals in industrialized nations with a high-income context and a cultural background similar to that of Germany tend to be rather skeptical about AI [22]. This skepticism also appears to extend to the use of AI in the health sector. Studies in such industrialized countries show that patients’ fear the risk of misdiagnosis when AI is used [23]. In a study by Nelson et al [24] on skin cancer diagnostics, patients expressed concerns about inadequate training sets in the development of AI. Distrust in the quality of AI modeling can lead to a significantly higher risk assessment of its use [25]. Transparency in the use of AI and disclosure to patients are seen as ways to promote acceptance [26]. The importance of such aspects for patients’ willingness to choose a radiologist who uses AI in situations that affect them personally has hardly been studied to date. Various studies have shown that patients are more supportive of AI roles where the technology supports—rather than replaces—physicians [27,28]. In the study by Jutzi et al [29], only 41% of patients advocated for AI as a stand-alone system in an online survey on skin tumor diagnostics, while the majority of 94% preferred AI solely to support the physician. The study by Ozcan et al [30] on mammography diagnostics showed similar results. Only 4% of patients accepted a stand-alone AI interpretation, while a majority of 71% preferred AI to be used as a second opinion. If the AI output is not checked by physicians, patients distrust the diagnosis more than they would if it were reviewed by a physician [31,32]. So far, however, there is little information about the reasoning behind patients’ observed mistrust of AI. The same applies to the role of the physician and the importance of the physician-patient relationship. Studies have merely shown that personal communication and empathy are considered necessary for a satisfactory physician-patient relationship and should not be endangered by the use of AI [33,34]. However, it remains unclear which characteristics of the physician-patient relationship can promote trust and to what extent these could be impaired in patients’ perceptions through the use of AI in radiological diagnostics.

The aim of this study was to fill these gaps by gaining insights into patients’ perspectives on risks and benefits, under what conditions patients accept the use of AI in radiological diagnostics and trust the AI diagnosis, what they expect from physicians, and what motives underlie their attitudes.

## Methods

### Study Design

A focus group design was selected for the study [35,36]. The inherent group dynamics of the focus group format allow for deep insights into the nature of sensitive and difficult-to-grasp topics, such as acceptance and trust regarding complex health-related technological developments [37]. This study used high-stakes personal health scenarios to capture often rather implicit assumptions, beliefs, values, and expectations. The study design and reporting followed the COREQ (Consolidated Criteria for Reporting Qualitative Research) guidelines (Checklist 1) [38].

### Data Collection

Patients were recruited with the help of patient organizations and self-help representatives who forwarded the invitation to participate in the study to their members, allowing them to register for the study on their own initiative. In addition, calls were made via social media, and flyers were distributed at relevant events. The participants were selected according to the principle of “purposive sampling” [39]. The inclusion criteria considered were that participants had undergone preventive medical checkups and/or were currently under the care of a general practitioner or specialist. They also needed to be proficient in German and at least the age of 18 years.

Focus groups were conducted from November 2022 to June 2023 in the state of North Rhine-Westphalia, Germany. They were conducted until saturation of arguments was reached [40]. Each focus group consisted of 4 to 6 participants. Participants were asked to complete a short questionnaire on demographic data, experiences with radiological examinations, and affinity for technology using the German version of the Affinity for Technology Interaction Scale [41]. All focus groups were held as face-to-face sessions. No relationship was established between participants and researchers prior to the focus groups. The participants were informed about the project’s objectives, the interdisciplinary research team, and the participating research institutions. The empirical study was conducted by a senior researcher (psychologist) with extensive experience in qualitative social research methods. She was assisted by a junior researcher (psychologist).

At the beginning of the focus groups, participants received standardized information on AI–based diagnostics in radiology (Multimedia Appendix 1), as well as information on the purpose and procedure of the focus group. A semistructured, pilot-tested guide served as the framework for the discussion in all focus groups. Prior to the study, interviews were conducted with representatives of patient organizations and medical professional associations to identify relevant topics and develop the guide in a manner appropriate for the target group. The interview guide comprised key questions and additional questions.

To start the discussion, the participants were asked the following questions: “What opportunities and risks do you see?” and “What would need to be guaranteed for you to endorse the use of AI in radiology? Are there circumstances under which you would reject its use?” In the main part of the discussion, key questions were introduced through personal involvement scenarios. The first scenario described the following situation: “Suppose your physician is unsure whether you are developing a serious illness, such as multiple sclerosis, and would like to refer you for an MRI scan to detect the disease as early as possible and, if necessary, initiate treatment.” Participants were then asked, “Would you choose a radiologist who does or does not use AI? What would your decision depend on? Which AI function would you feel most comfortable with when evaluating images or making a diagnosis?” The second scenario described a situation in which an MRI report had been generated using AI and contained indications of abnormalities that suggested the presence of a serious disease, such as multiple sclerosis. Participants were subsequently asked, “Would you trust the AI diagnosis? Under what circumstances? Who would you trust more, the physician or the AI, if your physician doubts the AI diagnosis?” In the third scenario, participants were asked to imagine a development in which AI is used extensively in diagnostics. The following key questions were asked, “Could this change your physician-patient relationship, or would it remain unaffected? What expectations do you have of your physician in relation to this future development?”

For all key questions, participants were encouraged to explain the reasons for their views. The key questions were visualized to stimulate thinking and argumentation. All focus groups were facilitated by the author in a neutral role. A “funnel design” was used [42]. In each group, 1 research assistant noted nonverbal information on the interaction among the participants. Each focus group lasted around 2 hours, was audiotaped, and transcribed verbatim.

### Data Analysis

Data analysis was performed using structured qualitative content analysis according to Mayring [43], as well as framework analysis [44]. Both methods are research techniques for the systematic analysis of manifest and latent communication content. The strength of structured qualitative content analysis lies in its theoretically grounded, rule-based categorization. When combined with matrix-based framework analysis, comparisons can be made across themes and cases, enabling patterns to be identified. This allows for a broad and in-depth analysis of the data, particularly when dealing with extensive datasets—as in this study.

Deductive and inductive categorization were combined using the software MAXQDA 2022 (VERBI Software GmbH). Deductive categories formed the basic framework, which was supplemented with inductive categories. Conflicts arising between the 2 approaches, such as redundancies and the resulting ambiguities in the category system, required revisions, standardizations, and re-examinations based on the raw data. The resolution of such conflicts was managed through a high degree of flexibility in the creation of the category system and transparent, traceable documentation of all disagreements and adjustments. Any disagreements arising during the development of categories were discussed within the research team until consensus was reached. In addition to the author, another coder from the research team was involved to ensure quality. There was good interrater reliability (κ=0.73).

In the first step, both raters read all transcripts, wrote brief summaries of what had been discussed, and made comments on the text passages that appeared relevant to them. In the second step, main thematic categories were developed deductively based on the literature review and the guide. A total of 4 focus groups were rated by the 2 raters independently of each other, the results were discussed, and the preliminary categories were modified and added to. The entire material was then rated based on the main categories. In the third step, the inductive development of subcategories was carried out. In several rounds of discussion, the entire category system was broken down, and the entire material was then rated independently. In the fourth step, statement matrices were organized according to topics, and, finally, documents were created on the basis of quotations.

### Ethical Considerations

This focus group study with patients was part of an extensive study that also included focus groups with physicians and a representative survey of the German population to investigate attitudes toward radiological diagnostics using AI. The study was approved by the Committee for Ethics in Research of the Forschungszentrum Jülich (FRAIM.20221005). Written informed consent was obtained from all participants prior to the empirical study. Data were pseudonymized and stored in a protected digital environment. Participants received €100 (US $117) for their participation.

## Results

### Overview

A total of 7 focus group sessions were conducted with 34 adults aged between 23 and 85 (mean 49.06, SD 17.08) years. Fifteen participants were female, and 19 were male. A majority (30/34, 88.2%) reported Abitur, the German equivalent of a high school diploma allowing university studies, as their highest level of general education. A total of 4 (11.8%) participants reported completing 10 years of formal education (German “Realschul-Abschluss, Mittlere Reife,” or equivalent). The majority of participants (25/34, 75.8%) were employed. Almost all reported having a regular family physician (32/34, 94.1%) and having undergone at least 1 radiological scan (32/33, 97%). The overall score for the 9-item Affinity for Technology in Action Scale indicated a relatively high affinity for technology among the sample (mean 4.14, SD 1.04; Cronbach α=0.94).

The following results highlight the opportunities and risks of using AI in radiological diagnostics from the patients’ perspective, as well as the circumstances that can foster its acceptance and trust. Citations of participants’ statements are provided with the focus group number (FG) and participant number (#). Original statements have been translated into English for publication.

The following aspects emerged during the focus group discussions: (1) efficiency, data integrity, and quality assurance; (2) data privacy, transparency, and explainability; (3) the role of physicians; (4) trust in physicians versus AI; (5) trust-building functions of physicians; and (6) the physician-patient relationship.

### Efficiency, Data Integrity, and Quality Assurance

In general, all participants agreed that the use of AI offers opportunities for more efficient and accurate diagnostics. However, a more efficient workflow and shorter reading times, thanks to AI, were only rated positively if these improvements shortened waiting times for appointments in radiology, enabled diseases to be detected earlier, and gave physicians more time for their patients.

The participants considered the potential of using AI to reduce human errors and achieve a high level of accuracy in diagnostics to be positive, particularly in the case of abnormalities that are difficult to identify:

*It is clearly an opportunity to diagnose things that would otherwise have been overlooked. Because AI never gets tired, so to speak, and makes fewer mistakes, depending on how it is trained*.

[FG3#1]

*Nevertheless, many participants expressed concern about misdiagnoses. Above all, they doubted the quality of the data, namely “that AI systems usually have a kind of bias, which is due to training, i.e. what the machine is fed with.”*

[FG6#2]

In addition, participants feared that the increasing commercialization of the AI market would lead to companies basing their actions purely on economic aspects. This could not only result in insufficient validation of data and discrimination against certain patient groups due to a lack of balance and diversity in the datasets, but also in algorithms being manipulated by companies to suit their own interests:

*I think the point is that if you end up with this kind of corporate competition, it could naturally lead to the problem of companies wanting to establish themselves that don't really have the know-how or experience. And then you run a bit of a risk because they then make dumping offers to university hospitals or radiology practices, and their software is then used simply because they offer the cheapest price, but it’s not good*.

[FG4#2]

Careful quality assurance, both in the development and approval of AI algorithms and in their ongoing application, was highlighted as one of the fundamental prerequisites for acceptance (Table 1). Participants emphasized that high data quality, in terms of scope, diversity, and continuous updating, is crucial and that this must be ensured through quality standards and regulation. They also acknowledged that science plays an important role in building trust in the quality of the algorithms. The participants considered it essential that the accuracy of diagnostics using AI is better than without AI:

*“AI has to be somewhat better at the end of the day, because otherwise it would make no sense at all—at least from the patient’s point of view.”*

[FG4#4]

Some participants found it contradictory to place higher demands on technology than on physicians in order to trust the technology.

*And what I keep asking myself is that we now always set this requirement of 98, 99 percent for technology, and then the initial question always arises: How high is the accuracy in humans? There are so many errors. So I ask myself why we are so strict with technology. So I don’t have an answer to that either. But I've noticed several times now that we simply set much higher standards for technology than we do for ourselves*.

[FG5#5]

**Table 1.** Framework conditions for the use of artificial intelligence (AI) in radiological diagnostics.

Category	Illustrative quote
Quality standards	“...That there is also a gold standard for how to set up such an AI or how it is fed with data.“ (FG6#4)
Validation studies	“...That I can rely on it. It must be validated.“ (FG6#6)
Independent certification	“The software must be independently certified in any case. (FG4#3)
Regulation	“The government and political organizations should definitely create the legal framework.“ (FG3#4)
Scientific monitoring	“That’s why it has to be scientifically monitored and constantly questioned: Are the results produced with this technology really still very close to the final result, the disease?“ (FG2#3)
Higher accuracy than radiologists	“I would want to have a high accuracy of 99 or so. Yes, already better than humans. It’s not just measured by accuracy. Humans can simply do more than AI as a whole package; they are not limited to the case. They have many, many more abilities, and that’s why it’s a factor, and I would want a little more than just saying, “Okay, AI is currently surpassing humans.” It should be significantly more than just a few percent better.“ (FG5#1)
Data protection	“So what would be important to me personally, for example, is that such data cannot be misused. Whether by employers, insurance companies, or third parties such as companies.“ (FG4#4)
Data security	“We’ve seen how hospitals have been hacked, how doctors’ offices have been hacked. I’m very, very skeptical about that.“ (FG3#3)
Freedom of choice	“And for me, it would be important that I am allowed to decide for myself, as long as I am able to: Will AI be used or not?“ (FG6#4)
Transparency	“How much data has been entered and so on, so that I can better assess it for myself. Someone would have to explain that to me.“ (FG6#4)
Explainability	“There is a good reason for using deep learning. The hit rates are the best. And even if I don’t understand it technically, I don’t care, as long as it works.“ (FG4#2) “When AI diagnoses things, it would be nice to know why and on what criteria it bases its diagnosis. Yes, I don’t know if I would trust it then.” (FG7#5)

### Data Privacy, Transparency, and Explainability

Most participants considered existing data protection in Germany to be sufficient for the use of AI in radiology. However, a minority of participants expressed extreme discomfort with the collection and storage of data for the development of AI and its application. These participants stressed the great importance of preserving the freedom of choice.

Ensuring transparency in the use of AI was important to all participants. As technical nonprofessionals, the participants did not feel able to assess the quality of AI in diagnostics. Given the potentially serious consequences of misdiagnosis, the types of training data used should be disclosed, and the accuracy of the algorithm should be specified and clearly communicated. The reputation of the companies that develop the software was considered a good indicator of the software’s trustworthiness. Although transparency was seen as a necessary prerequisite for the acceptance of AI use, participants were unsure whether this would help build trust in AI diagnoses if they were personally affected.

Participants expressed contrasting attitudes toward the explainability of AI diagnoses. One group considered the “black box” nature of AI to be unproblematic as long as superior performance was proven. This argument did not convince the roughly equally large group that found the “black box” nature of AI problematic. For these participants, the process by which AI arrives at a result must be reconstructible in order to trust an AI diagnosis, clarify misdiagnoses, and assign responsibility.

The framework conditions that patients demanded for the use of AI in radiological diagnostics are listed in Table 1. The quotes are typical examples of the respective categories.

### Role of Physicians

Meeting the technical requirements and framework conditions for the use of AI does not automatically lead to a willingness to choose a radiologist who uses AI.

In a situation where a suspected serious neurological disease, such as multiple sclerosis, needs to be clarified radiologically, all but 3 participants indicated that they would choose a radiology practice that uses AI. However, this preference depended on the interpretation and evaluation of the clinical relevance of abnormalities and the final diagnosis remaining the responsibility of radiologists in consultation with the referring physicians.

With the exception of 1 person, who preferred automated AI in all cases, the participants felt strongly that AI should only be used as a tool in the hands of an experienced physician:

*The decision on how to interpret this, how relevant it is to the individual case, and how to deal with it must remain with the physician, without restriction*.

[FG2#5]

### Trust in Physicians Versus AI

The participants considered an independent medical assessment to be important for trusting the AI diagnosis. This was particularly evident in cases where the attending physician doubted the AI’s diagnosis.

If abnormalities were found that required treatment, the AI diagnosis did not automatically lead to a willingness to undergo treatment. Only 1 participant mentioned that they would trust the AI diagnosis and follow the treatment. On the other hand, only a minority trusted the physicians’ judgment unreservedly in such cases. The majority pursued a safety strategy and wanted to eliminate uncertainty by seeking a third opinion. An AI-supported procedure could also be used again, but in the vast majority of cases, a physician was considered necessary to review the AI result again.

### Trust-Building Functions of Physician

One of the reasons for the important role of the physician is the requirement to check the AI output. Many participants demanded not only a plausibility check but also a verification of the AI output through an additional independent assessment. For most participants, the decisive factors were characteristics and skills that were attributed only to physicians and considered indispensable for ensuring well-balanced results based on individual cases (Table 2). In their opinion, physicians have a holistic view of human beings, which enables them to classify the clinical picture in terms of the patient’s entire biography and take into account individual factors that may be relevant to the diagnosis.

**Table 2.** Trust-building functions of the physician

Category	Illustrative quote
Checks to prevent misdiagnoses	“I see the risk that the machine will still make mistakes and that someone will still have to check whether the diagnosis is actually correct.” (FG5#2)
Backup through independent diagnostics	“…On the one hand, you want to secure support through AI, but on the other hand, you cannot rely on it 100%. Instead, the doctor himself must evaluate the images each time and form his own opinion.” (FG4#2)
Taking the individuality of the patient into account	“For me, the doctor is still the one who understands the human system as a whole. And AI is just the tin idiot that evaluates data and draws conclusions from it. So AI always has only one focus: please evaluate this image data now. And spit out the results.” (FG3#4)
Expanding the diagnostic context through intuition, flexibility, and creativity	“…That something so holistic and inventive is simply not achievable with AI. The creativity that is inherent in humans and that is ultimately needed when making a diagnosis.” (FG5#1)

It was also argued that physicians can use their intellectual abilities to deviate from routines. Their intuition, creativity, and flexibility enable them to do so. It is precisely these qualities of physicians that are highly valued for recognizing abnormalities beyond the specific issue at hand and for diagnosing rare diseases.

At the same time, however, participants were skeptical that the trust-building functions of physicians would be realized. In their view, the use of AI could lead to a decline in the competence of radiologists. The greatest risks were seen in physicians’ overconfidence in AI output and insufficient attention to, and monitoring of, the results:

*Of course, this will lead to a decline in quality, because doctors throughout the entire chain will rely on AI, and things will simply be checked off more quickly: AI gives this and that diagnosis, take another look: Yes, it’s okay. If the attending physician gets that, he says: Yes, the other doctor has already given his okay, so everything is fine and we'll do it that way*.

[FG5#2]

In addition, the participants assumed that the combination of AI and radiologists is only a transitional stage toward automated AI. The participants viewed this development with great concern:

*Because I see a bit of a timeline there. I think that as soon as AI comes into play, sooner or later it will come down to the third point: automated AI. Because maybe at the beginning you trust the radiologist more because they have more experience. Then AI has proven itself so many times that you think, okay, AI has the final say*.

[FG3#6]

### Physician-Patient Relationship

It was important to the participants that the social and emotional aspects of the physician-patient relationship were preserved (Table 3). This is not just about communication but rather about experiencing empathy, a reciprocal exchange relationship, and the feeling of being in good hands. It was crucial for participants to know that physicians would continue to take responsibility for their patients even when using AI. It is important to them that physicians make decisions based on the well-being of their patients. For patients, this means that physicians act not only on the basis of their knowledge but also on the basis of values. In addition, physicians should explain to patients what AI is, how it is used, how its results should be evaluated, and, above all, what the added value of an AI-supported diagnosis is.

Most participants were concerned about the threat that the physician-patient relationship might suffer:

*I can see the danger of this becoming the norm in practices. The AI result is there: bang, let’s do it, move on. So the doctor might take less time for a personal consultation and no longer look closely at the patient*.

[FG2#2]

They were skeptical that relieving physicians’ workloads through greater efficiency using AI would actually result in more time for patients. Rather, they fear that dehumanization and deindividualization in health care will increase.

**Table 3.** Trust-building effects of the physician-patient relationship.

Category	Illustrative quote
Creating an interpersonal relationship	“For me, it would be incredibly important to be with the doctor and see how he takes a close look at it again. And it’s very, very, very important that the patient feels well cared for at that moment and doesn’t just have a piece of paper in their hand.” (FG1#1)
Taking responsibility for the patient	“For me, the risk basically lies in a machine making diagnoses based on the facts that my body provides or that the disease shows. But there is more to it than that. Responsibility also plays a role here.” (FG1#3)
Making decisions based on moral values	“For me, AI stands for logic. And for me, the doctor stands for conscience or human traits and for hesitation. So not in a way like: one or zero, one or zero.” (FG7#3)
Explaining the individual added value of using AI^a^	“I mean, when patients arrive and say, ‘Yes, why are you doing that?,’ then the doctor must at least be able to explain why he is doing it and what the advantages are, and also what the patient will gain from it.” (FG2#4)

a AI: artificial intelligence.

## Discussion

### Principal Findings

The results of this study show that patients are generally open to the use of AI in radiological diagnostics. This is consistent with a number of other studies that demonstrate openness to AI in other medical fields among various population groups [45,46]. Patients recognize the potential for more accurate, faster diagnostics based on expanded medical knowledge, while emphasizing the importance of quality assurance not only during approval but also during ongoing application.

However, the in-depth discussion in this study shows that the advantages mentioned will only increase acceptance of AI if, at the same time, the health care system is set up in such a way that the use of AI leads to real added value for patients. Patient-centered care was one of the most important factors that need to be met for patients to endorse the use of AI. In particular, this means that physicians should use the time saved through the use of AI to provide more personalized care for patients. Rationalization effects and cost savings—such as faster patient flow (ie, more patients in the same amount of time), staff savings, and even the replacement of radiologists by AI—were major reasons why patients would reject its use. Furthermore, the expected increasing commercialization of AI development and application was associated with negative impacts on patient care. These aspects have received too little attention in previous studies.

This study suggests that it is not only technological features—in particular, high performance and reliability—that are decisive for the acceptance of AI; rather, the quality of the entire sociotechnical system plays a role.

This is particularly evident when it comes to the specific willingness to choose a radiologist who uses AI if there is a suspicion of a serious disease that requires radiological clarification. Patients are uncertain in this regard. In such a situation, patients place high demands on the role and function of physicians in particular. As already shown in other studies, it is very important to patients that AI findings are checked by physicians [47,48].

Additionally, this study was able to expand previous findings by showing that what is required is not merely a plausibility check, but rather an independent diagnosis that broadens the diagnostic context. The question is not whether AI is used as a first or second opinion; rather, physicians should contribute specific skills and competencies to the diagnostic process that, from the patients’ perspective, AI lacks, such as intuition, flexibility, and creativity. Only then, in the participants’ view, will the individuality of each case be given appropriate consideration and abnormalities beyond the specific question for which the AI had been trained will be discovered. The inability of AI to integrate isolated imaging results within the broader context of the patient’s living situation is also discussed in the radiology community as a significant limitation of today’s AI systems. It is frequently emphasized that the more nuanced expertise of radiologists is indispensable in the diagnostic process [49].

However, patients are concerned that these very functions of the physician, which promote trust, could be weakened by overreliance on AI results and the increasing deskilling of physicians. This is all the more concerning as the trend toward automated AI is both expected and feared. This contrasts with the fact that radiologists themselves consider the replacement of radiologists unlikely [50].

The results illustrate that patients are highly uncertain about whether they would actually opt for AI diagnostics if they were personally affected. This is also indicated by the finding that patients tend to trust physicians more than AI results if the physician doubts the AI results.

The results of this study show that it is not only the competence of physicians that matters, but also the physician-patient relationship. This result confirms previous findings that the maintenance of the familiar physician-patient relationship is decisive for patients [51,52]. The in-depth discussions in the focus groups clarified which characteristics of the physician-patient relationship are important when using AI. An empathetic relationship and the physician’s responsibility to care for the patient’s well-being are required. In addition, patients emphasized that physicians should take on an advisory role with regard to the pros and cons of using AI in individual cases. This also includes being able to provide information about the quality of AI. Various studies have shown the importance of transparency in the use of AI and the desire of patients to be informed about whether AI is being used [53,54]. The results of this study reveal, in addition, that physicians, as trusted mediators of information, should play a central role in dispelling concerns and fears. These factors in the physician-patient relationship could build trust in the diagnostic process. Trust can promote acceptance of the use of AI [55,56]. However, AI also has the potential to disrupt person-centered physician-patient relationships [57,58]. This study demonstrated that this is precisely what patients are concerned about when it comes to the use of AI in radiological diagnostics.

The findings of the study, and in particular the important role of the physician-patient relationship, arise within the context of Germany’s highly developed health care system, which is characterized by high standards. As a rule, equal access to new technologies is guaranteed, with costs being shared within the system in accordance with the principle of solidarity. Ethical and regulatory considerations are taken into account prior to the implementation of new technologies. In health care systems in other countries that are structured differently, patients might consider other factors to be relevant for acceptance and trust, or weigh them differently.

### Strengths and Limitations

This study examined patients’ perspectives on the use of AI in radiological diagnostics using an innovative focus group method. Unlike many previous studies, which tended to use a more abstract approach, this study used scenarios as stimuli to gain insights into patients’ reasoning regarding the use of AI in situations that affect them personally. This approach expands current research by focusing on patients’ motives in their decisions for or against a radiologist who uses AI. A limitation of the study could be that the patients had a high level of education and a strong affinity for technology. Both of these factors could lead to a more positive attitude toward AI compared to the general population [59]. Although several channels were used to reach participants and no prior knowledge of AI was required for participation, self-selection and recruitment via patient organizations and self-help groups may have introduced some selection bias. Therefore, the results may have limited applicability to patients with a low socioeconomic status and low digital and health literacy. However, this does not detract from the validity of the argumentation patterns identified in this qualitative study. Nevertheless, validating the results with a more heterogeneous sample in terms of socioeconomic status and digital literacy might further strengthen the conclusions.

### Conclusions

This study shows that patients are generally open to the use of AI in radiological diagnostics, depending on the design of the sociotechnical system. It is not only the performance of AI that is decisive; extensive quality assurance measures are necessary, as is, above all, the integration of AI into the health care system in a way that ensures real added value for patients. At the same time, the study reveals that in cases of personal involvement, the decision to use AI—beyond the perceived abstract advantages and disadvantages—depends significantly on the trust-building role of the physician in the diagnostic process. Consequently, framework conditions for the use of AI are required that avoid constraints preventing physicians from acting independently and degrading them to “messengers of AI.” Overconfidence in the AI result and deskilling must be prevented. The integrity of the physician-patient relationship should be preserved. On this basis, patients do not feel “at the mercy” of AI but rather “in good hands” in the diagnostic process guided by the physician. This can help to overcome existing uncertainties. Acceptance and trust can be promoted by strengthening the trust-building role of physicians when AI is used.

## Supplementary material

10.2196/89178 Multimedia Appendix 1 Short introduction to artificial intelligence (AI) given to participants at the beginning of the focus group discussions.

10.2196/89178 Checklist 1 COREQ checklist.

## Abbreviations

AI: artificial intelligence
COREQ: Consolidated Criteria for Reporting Qualitative Research
MRI: magnetic resonance imaging

## References

[1] Alowais SA, Alghamdi SS, Alsuhebany N (2023). Revolutionizing healthcare: the role of artificial intelligence in clinical practice. BMC Med Educ.

[2] Najjar R (2023). Redefining radiology: a review of artificial intelligence integration in medical imaging. Diagnostics (Basel).

[3] Pinto-Coelho L (2023). How artificial intelligence is shaping medical imaging technology: a survey of innovations and applications. Bioengineering (Basel).

[4] Khalifa M, Albadawy M (2024). AI in diagnostic imaging: revolutionising accuracy and efficiency. Comput Methods Programs Biomed Update.

[5] Wang H, Zhu H, Ding L (2022). Accurate classification of lung nodules on CT images using the TransUnet. Front Public Health.

[6] Nam JG, Hwang EJ, Kim J (2023). AI improves nodule detection on chest radiographs in a health screening population: a randomized controlled trial. Radiology.

[7] Nabizadeh F, Masrouri S, Ramezannezhad E (2022). Artificial intelligence in the diagnosis of multiple sclerosis: a systematic review. Mult Scler Relat Disord.

[8] Topol EJ (2019). High-performance medicine: the convergence of human and artificial intelligence. Nat Med.

[9] Younger J, Morris E, Arnold N, Athulathmudali C, Pinidiyapathirage J, MacAskill W (2026). A systematic review of comparisons of AI and radiologists in the diagnosis of HCC in multiphase CT: implications for practice. Jpn J Radiol.

[10] Becker J, Decker JA, Römmele C (2022). Artificial intelligence-based detection of pneumonia in chest radiographs. Diagnostics (Basel).

[11] Aljuhani M, Ashraf A, Edison P (2024). Use of artificial intelligence in imaging dementia. Cells.

[12] Cross JL, Choma MA, Onofrey JA (2024). Bias in medical AI: implications for clinical decision-making. PLOS Digit Health.

[13] Hasanzadeh F, Josephson CB, Waters G, Adedinsewo D, Azizi Z, White JA (2025). Bias recognition and mitigation strategies in artificial intelligence healthcare applications. NPJ Digit Med.

[14] Koçak B, Ponsiglione A, Stanzione A (2025). Bias in artificial intelligence for medical imaging: fundamentals, detection, avoidance, mitigation, challenges, ethics, and prospects. Diagn Interv Radiol.

[15] Salahuddin Z, Woodruff HC, Chatterjee A, Lambin P (2022). Transparency of deep neural networks for medical image analysis: a review of interpretability methods. Comput Biol Med.

[16] Hassija V, Chamola V, Mahapatra A (2024). Interpreting black-box models: a review on explainable artificial intelligence. Cogn Comput.

[17] Dratsch T, Chen X, Rezazade Mehrizi M (2023). Automation bias in mammography: the impact of artificial intelligence BI-RADS suggestions on reader performance. Radiology.

[18] Ling Kuo RY, Freethy A, Smith J (2024). Stakeholder perspectives towards diagnostic artificial intelligence: a co-produced qualitative evidence synthesis. EClinicalMedicine.

[19] Carter SM, Carolan L, Saint James Aquino Y (2023). Australian women’s judgements about using artificial intelligence to read mammograms in breast cancer screening. Digit Health.

[20] Fukuyama F (1995). Trust: The Social Virtues and the Creation of Prosperity.

[21] Wang S (2025). Public perceptions of artificial intelligence in 20 countries: assessing individual- and country-level factors. Cross Cult Res.

[22] Howell B (2025). WEIRD? Institutions and consumers’ perceptions of artificial intelligence in 31 countries. AI Soc.

[23] Gatting L, Ahmed S, Meccheri P, Newlands R, Kehagia AA, Waller J (2024). Acceptability of artificial intelligence in breast screening: focus groups with the screening-eligible population in England. BMJ Public Health.

[24] Nelson CA, Pérez-Chada LM, Creadore A (2020). Patient perspectives on the use of artificial intelligence for skin cancer screening: a qualitative study. JAMA Dermatol.

[25] Esmaeilzadeh P (2020). Use of AI-based tools for healthcare purposes: a survey study from consumers’ perspectives. BMC Med Inform Decis Mak.

[26] Tang L, Li J, Fantus S (2023). Medical artificial intelligence ethics: a systematic review of empirical studies. Digit Health.

[27] Young AT, Amara D, Bhattacharya A, Wei ML (2021). Patient and general public attitudes towards clinical artificial intelligence: a mixed methods systematic review. Lancet Digit Health.

[28] Osnat B (2025). Patient perspectives on artificial intelligence in healthcare: a global scoping review of benefits, ethical concerns, and implementation strategies. Int J Med Inform.

[29] Jutzi TB, Krieghoff-Henning EI, Holland-Letz T (2020). Artificial intelligence in skin cancer diagnostics: the patients’ perspective. Front Med (Lausanne).

[30] Ozcan BB, Dogan BE, Xi Y, Knippa EE (2025). Patient perception of artificial intelligence use in interpretation of screening mammograms: a survey study. Radiol Imaging Cancer.

[31] Rodler S, Kopliku R, Ulrich D (2024). Patients’ trust in artificial intelligence-based decision-making for localized prostate cancer: results from a prospective trial. Eur Urol Focus.

[32] Fransen SJ, Kwee TC, Rouw D (2025). Patient perspectives on the use of artificial intelligence in prostate cancer diagnosis on MRI. Eur Radiol.

[33] Miró Catalina Q, Femenia J, Fuster-Casanovas A (2023). Knowledge and perception of the use of AI and its implementation in the field of radiology: cross-sectional study. J Med Internet Res.

[34] Akingbola A, Adeleke O, Idris A, Adewole O, Adegbesan A (2024). Artificial intelligence and the dehumanization of patient care. J Med Surg Public Health.

[35] Willig C, Stainton-Rogers W (2017). The SAGE Handbook of Qualitative Research in Psychology.

[36] Ryan KE, Gandha T, Culbertson MJ, Carlson C Focus group evidence: implications for design and analysis. Am J Eval.

[37] Morgan DL, Gubrium JF, Holstein JA, Marvasti AB, McKinney KD (2012). The Sage Handbook of Interview Research: The Complexity of the Craft.

[38] Tong A, Sainsbury P, Craig J (2007). Consolidated criteria for reporting qualitative research (COREQ): a 32-item checklist for interviews and focus groups. Int J Qual Health Care.

[39] Robinson RS, Michalos AC (2014). Encyclopedia of Quality of Life and Well-Being Research.

[40] Hennink M, Kaiser BN (2022). Sample sizes for saturation in qualitative research: a systematic review of empirical tests. Soc Sci Med.

[41] Franke T, Attig C, Wessel D (2019). A personal resource for technology interaction: development and validation of the affinity for technology interaction (ATI) scale. Int J Hum Comput Interact.

[42] Morgan DL, Krueger RA, King JA (1998). The Focus Group Guidebook.

[43] Mayring P (2014). Qualitative content analysis theoretical background, recent developments and software solutions. https://tinyurl.com/m75xxr22.

[44] Ritchie J, Spencer L, O’Connor W, Ritchie J, Lewis J (2003). Qualitative Research Practice: A Guide for Social Science Students and Researchers.

[45] Scott IA, Carter SM, Coiera E (2021). Exploring stakeholder attitudes towards AI in clinical practice. BMJ Health Care Inform.

[46] Hogg HDJ, Al-Zubaidy M, Technology Enhanced Macular Services Study Reference Group (2023). Stakeholder perspectives of clinical artificial intelligence implementation: systematic review of qualitative evidence. J Med Internet Res.

[47] Riedl R, Hogeterp SA, Reuter M (2024). Do patients prefer a human doctor, artificial intelligence, or a blend, and is this preference dependent on medical discipline? Empirical evidence and implications for medical practice. Front Psychol.

[48] Lennartz S, Dratsch T, Zopfs D (2021). Use and control of artificial intelligence in patients across the medical workflow: single-center questionnaire study of patient perspectives. J Med Internet Res.

[49] Katal S, York B, Gholamrezanezhad A (2024). AI in radiology: from promise to practice - a guide to effective integration. Eur J Radiol.

[50] Yang L, Ene IC, Arabi Belaghi R, Koff D, Stein N, Santaguida PL (2022). Stakeholders’ perspectives on the future of artificial intelligence in radiology: a scoping review. Eur Radiol.

[51] Vo V, Chen G, Aquino YSJ, Carter SM, Do QN, Woode ME (2023). Multi-stakeholder preferences for the use of artificial intelligence in healthcare: a systematic review and thematic analysis. Soc Sci Med.

[52] Ibba S, Tancredi C, Fantesini A (2023). How do patients perceive the AI-radiologists interaction? Results of a survey on 2119 responders. Eur J Radiol.

[53] Kühne S, Jacobsen J, Legewie N, Dollmann J (2025). Attitudes toward AI usage in patient health care: evidence from a population survey vignette experiment. J Med Internet Res.

[54] McGhee KN, Barrett DJ, Safarini O (2026). Patient preferences for artificial intelligence in medical imaging: a single-center cross-sectional survey. J Imaging Inform Med.

[55] Afroogh S, Akbari A, Malone E, Kargar M, Alambeigi H (2024). Trust in AI: progress, challenges, and future directions. Humanit Soc Sci Commun.

[56] Evans RP, Bryant LD, Russell G, Absolom K (2024). Trust and acceptability of data-driven clinical recommendations in everyday practice: a scoping review. Int J Med Inform.

[57] Sauerbrei A, Kerasidou A, Lucivero F, Hallowell N (2023). The impact of artificial intelligence on the person-centred, doctor-patient relationship: some problems and solutions. BMC Med Inform Decis Mak.

[58] Zondag AGM, Rozestraten R, Grimmelikhuijsen SG (2024). The effect of artificial intelligence on patient-physician trust: cross-sectional vignette study. J Med Internet Res.

[59] Busch F, Hoffmann L, Xu L (2025). Multinational attitudes toward AI in health care and diagnostics among hospital patients. JAMA Netw Open.

