# Supplementary material for: Patients’ Perspectives on the Implementation of AI in Radiological Diagnostics: Focus Group Study
Source: J Med Internet Res. 2026 May 25;28:e89178. doi: 10.2196/89178 (PMC13200804; doi:10.2196/89178)
Supplement: Multimedia Appendix 1 [file jmir-v28-e89178-s001.docx]

**Multimedia Appendix**

Short introduction to AI given to participants at the beginning of the focus group discussions.

AI is a technology that aims to replicate human mental abilities. In radiology, AI is a technology that is being developed for specific tasks, primarily pattern recognition in image material (e.g., from CT or MRI scans) for diagnostic purposes. AI can also be used for early detection of diseases and monitoring of disease progression. How can AI achieve this? The AI software program is fed with learning material consisting of image data from radiology and the corresponding diagnoses by the radiologist. This involves many thousands of data sets. The program learns to distinguish between clinically abnormal and normal images. After completing the training phase, the AI program is able to independently process new, unknown images and recognize whether certain diseases are present or not. Such diagnoses could be the presence of cancer tumors or diseases such as multiple sclerosis. So far, there are only a few radiology practices that already use AI, but in the future, this technology could become widespread.
