# Supplementary material for: Patients’ Perspectives on the Implementation of AI in Radiological Diagnostics: Focus Group Study
Source: J Med Internet Res. 2026 May 25;28:e89178. doi: 10.2196/89178 (PMC13200804; doi:10.2196/89178)
Supplement: Checklist 1 [file jmir-v28-e89178-s002.docx]

COREQ checklist

Consolidated criteria for reporting qualitative studies (COREQ): 32-item checklist

Developed from:

Tong A, Sainsbury P, Craig J. Consolidated criteria for reporting qualitative research (COREQ): a 32-item checklist for interviews and focus groups. International Journal for Quality in Health Care. 2007. Volume 19, Number 6: pp. 349 – 357

| **Item** **No** | **Guide** **Questions/** **Description** | **Reported** **on** **Page** **#** |
| --- | --- | --- |
| **Domain** **1:** **Research** **team** **and** **reflexivity** | | |
| **Personal** **Characteristics** | | |
| 1. Interviewer/ facilitator | Which author/s conducted the interview or focus group?  **The author of the manuscript, supported by a member of the research team** | Page 3 |
| 2. Credentials | What were the researcher’s credentials? E.g., PhD, MD  **Certified Psychologists.** | Page 3 |
| 3. Occupation | What was their occupation at the time of the study?  **Senior researcher, the other as junior researcher**. | Page 3 |
| 4. Gender | Was the researcher male or female?  **Both female**. |  |
| 5. Experience and training | What experience or training did the researcher have?  **Various previous projects with qualitative methods of social research**. | Page 3 |
| **Relationship** **with** **participants** | | |
| 6. Relationship established | Was a relationship established prior to study commencement?  **No**. | Page 3 |
| 7. Participant knowledge of the interviewer | What did the participants know about the researcher? e.g. personal goals, reasons for doing the research?  **The letter of invitation which was sent to each participant explained the purpose of the study**. | Page 3 |
| 8. Interviewer characteristics | What characteristics were reported about the  interviewer/facilitator? e.g. Bias, assumptions, reasons and interests in the research topic  **The research focus and the backgrounds of the research team were reported**. | Page 3 |

| **Item** **No** | **Guide** **Questions/** **Description** | **Reported** **on** **Page** **#** |
| --- | --- | --- |
| **Domain** **2:** **study** **design** | | |
| **Theoretical framework** | | |
| 9. Methodological orientation and Theory | What methodological orientation was stated to underpin the study? e.g. grounded theory, discourse analysis, ethnography, phenomenology, content analysis  **Structured qualitative content analysis, framework analysis** | Page 3-4 |
| **Participant** **selection** | | |
| 10. Sampling | How were participants selected? e.g., purposive, convenience, consecutive, snowball  **By purposive sampling** | Page 3 |
| 11. Method of approach | How were participants approached? e.g., face-to-face, telephone, mail, email  **By email, social media, flyer** | Page 3 |
| 12. Sample size | How many participants were in the study?  **34 participants** | Page 4 |
| 13. Non-participation Setting | How many people refused to participate or dropped out? Reasons?  **None of the participants dropped out during the study.** |  |
| 14. Setting of data collection | Where was the data collected? e.g., home, clinic, workplace  **Neutral, conference rooms with quiet environment**. |  |
| 15. Presence of nonparticipants | Was anyone else present besides the participants and researchers?  **No** |  |
| 16. Description of sample | What are the important characteristics of the sample? e.g. demographic data, date  **Described in detail** | Page 4 |
| **Data** **collection** | | |
| 17. Interview guide | Were questions, prompts, and guides provided by the authors? Was it pilot tested?  **Yes** | Page 3,  Appendix 1 |
| 18. Repeat interviews | Were repeat interviews carried out? If yes, how many?  **No** |  |
| 19. Audio/visual recording | Did the research use audio or visual recording to collect the data?  **Audio** | Page 3 |
| 20. Field notes | Were field notes made during and/or after the interview or focus group?  **Yes** | Page 3 |
| 21. Duration | What was the duration of the interviews or focus group?  **Approximately 120 minutes**. | Page 3 |
| 22. Data saturation | Was data saturation discussed?  **Yes** | Page 3 |

| **Item** **No** | **Guide** **Questions/** **Description** | **Reported** **on** **Page** **#** |
| --- | --- | --- |
| 23. Transcripts returned | Were transcripts returned to participants for comment and/or correction?  **No** |  |
| **Domain** **3:** **analysis** **and** **findings** | | |
| **Data** **analysis** | | |
| 24. Number of data coders | How many data coders coded the data?  **Two** | Page 3 |
| 25. Description of the coding tree | Did the authors provide a description of the coding tree?  **Main topics and categories** | Page 5-7 |
| 26. Derivation of themes | Were themes identified in advance or derived from the data?  **Both (deductive and inductive)** | Page 3 |
| 27. Software | What software, if applicable, was used to manage the data?  **MAXQDA 2022** | Page 3 |
| 28. Participant checking | Did participants provide feedback on the findings?  **No** |  |
| **Reporting** | | |
| 29. Quotations presented | Were participant quotations presented to illustrate the  themes/findings? Was each quotation identified? e.g., participant number  **Yes** | Page 4-7 |
| 30. Data and findings consistent | Was there consistency between the data presented and the findings?  **Yes** |  |
| 31. Clarity of major themes | Were major themes clearly presented in the findings?  **Yes** | Page 4-7 |
| 32. Clarity of minor themes | Is there a description of diverse cases or a discussion of minor themes?  **Yes** | Page 5-6 |
